# Outcomes related to 4864 pregnancies with exposure to lopinavir/ritonavir (LPV/r)

**DOI:** 10.7448/IAS.17.4.19613

**Published:** 2014-11-02

**Authors:** Pat Tookey, Claire Thorne, Marisol Martinez-Tristani, Michael Norton, Jean van Wyk

**Affiliations:** 1 UCL Institute of Child Health, University College London, London, UK; 2 Global Pharmaceutical Research and Development, AbbVie, Inc, North Chicago, IL, USA

## Abstract

**Introduction:**

During pregnancy, LPV/r is a common anchor drug employed to treat the mother's HIV-1 infection in addition to reducing the risk of mother-to-child transmission (MTCT). The National Study of HIV in Pregnancy and Childhood (NSHPC) conducts a comprehensive population-based surveillance of HIV infection in pregnant women exposed to antiretroviral therapy (ART) in the UK and Ireland; in 2003–2012 over a third of pregnancies reported to the NSHPC involved exposure to LPV/r.

**Methods:**

We undertook a retrospective were descriptive analysis of individual NSHPC patient data, using pregnancy as the unit of observation. Clinical outcomes for pregnancies reported by June 2013, where women were exposed to LPV/r and due to deliver between January 2003 and December 2012, are described.

**Results:**

A total of 4864 LPV/r exposed pregnancies in 4118 women were identified. These resulted in 4702 deliveries with 4759 live and 46 stillborn infants. Seventy five percent of women were born in sub-Saharan Africa, 13% in the UK or Ireland. Median maternal age at conception was 30 years. Nine hundred and eighty (20%) pregnancies were conceived while taking LPV/r, with a median duration of LPV/r exposure of 270 days. A total of 3884 (80%) pregnancies initiated LPV/r after conception, with a median duration of LPV/r exposure of 107 days. Viral load (VL) close to delivery was available for 4083/4702 (87%) deliveries, with VL <50 c/mL in 73% and <1000 c/mL in 94% of women. VL by timing of LPV/r initiation is shown in Table 1. Sixty three percent of deliveries were by C-section, of which 62% were classified as elective and 38% as emergency. Among singleton liveborn infants, 13% were born prior to 37 weeks gestation (2.5% <32 weeks) and 15% had birth weight <2500 g (2.3% <1500 g). HIV infection status was available for 4039 (89%) singleton infants. For the periods 2003–2007 and 2008–2012, MTCT rates were 1.1% (95% CI 0.6–1.6) and 0.5% (95% CI 0.2–0.8) respectively. Hundred and thirty four live born children (2.8%) had at least one congenital abnormality reported.

**Conclusions:**

In the NSHPC database, in women exposed to LPV/r during pregnancy in the UK and Ireland, MTCT rates are low and continue to decline, and are similar to rates in the entire NSHPC cohort of women with diagnosed HIV [1]. The congenital abnormality rate is comparable with that reported for the uninfected population in this geographic region.

**Table 1:** Viral load close to delivery, by timing of LPV/r initiation

	Conceived on LPV/r (n=747)	Initiated LPV/r during pregnancy (n=3336)	Total (n=4083)
VL <50 c/mL	91% (n=677)	69% (n=2302)	73% (n=2379)
VL <1000 c/mL	98% (n=729)	93% (n=3100)	94% (n=3829)
